# The Eye That Hid Its Wound: A Case Report of Corneal Perforation Despite a Negative Seidel Test

**DOI:** 10.7759/cureus.111605

**Published:** 2026-06-27

**Authors:** Vindhya Prakash, Nishanth S, Kashish Goyal

**Affiliations:** 1 Ophthalmology, Shamnur Shivashankarappa Institute of Medical Sciences and Research Centre, Davangere, IND; 2 Ophthalmology, Shri Dharmasthala Manjunatheshwara College of Medical Sciences and Hospital, Bengaluru, IND

**Keywords:** blunt ocular trauma, corneal perforation, lens capsule rupture, negative siedel's test, ocular trauma, open globe injury

## Abstract

A 58-year-old man presented with pain, redness, watering, and sudden diminution of vision in the right eye following blunt trauma from a stone. Slit-lamp examination revealed mild corneal edema, a central corneal defect, a traumatic cataract with probable anterior lens subluxation, and a distinct air bubble within the anterior chamber. Seidel’s test was negative, and no iris prolapse or obvious corneal laceration was identified. The presence of intracameral air raised a strong suspicion of prior communication between the anterior chamber and the external environment, suggestive of a self-sealed corneal perforation, probably secondary to countercoup forces generated by blunt trauma. B-scan ultrasonography demonstrated an attached retina in all quadrants. Prompt surgical management was undertaken, resulting in restoration of globe integrity and stabilization of the ocular condition. Occult open-globe injuries following blunt trauma may present without an overt wound leak or classic signs of perforation. An isolated intracameral air bubble should alert clinicians to the possibility of a self-sealed globe injury, even in the setting of a negative Seidel test. Careful anterior segment evaluation and timely intervention are essential to prevent vision-threatening complications and preserve ocular integrity.

## Introduction

Ocular trauma remains one of the leading causes of monocular blindness and vision loss worldwide, particularly among working-age individuals [1]. The impact of ocular trauma has a devastating effect on the individual and can also burden the socioeconomic and healthcare resources of a country [2]. The Birmingham Eye Trauma Terminology (BETT) classification categorizes globe injuries as either closed or open. Open-globe injuries are further divided into ruptures and lacerations. While open-globe injuries commonly present with obvious clinical signs such as aqueous leakage, iris prolapse, or hypotony, certain injuries may remain deceptively occult because of spontaneous wound sealing. Such cases pose a significant diagnostic challenge and may lead to delayed intervention and poor visual outcomes.

Blunt trauma can occasionally generate sufficient shock waves to produce partial- or full-thickness corneal disruption without a retained intraocular foreign body or overt penetrating injury. In these situations, subtle clinical findings become critically important. One such underrecognized sign is the presence of an intracameral air bubble in the absence of prior ocular surgery or external introduction of air. This finding may indicate transient globe penetration with spontaneous sealing of the wound.

Traumatic cataract associated with an anteriorly subluxated lens further suggests significant transmission of force through the anterior segment structures. We report a rare and diagnostically intriguing case of suspected self-sealed corneal perforation with traumatic cataract following blunt trauma from a stone, in which the presence of a trapped air bubble within the anterior chamber served as a key clue to identifying an occult open-globe injury despite a negative Seidel test.

## Case presentation

A 58-year-old man presented to the emergency department with complaints of sudden painful diminution of vision, redness, watering, and swelling of the right eye following blunt trauma from a stone. There was no history of loss of consciousness, prior ocular surgery, spectacle use, or similar ocular complaints.

**Figure 1:**
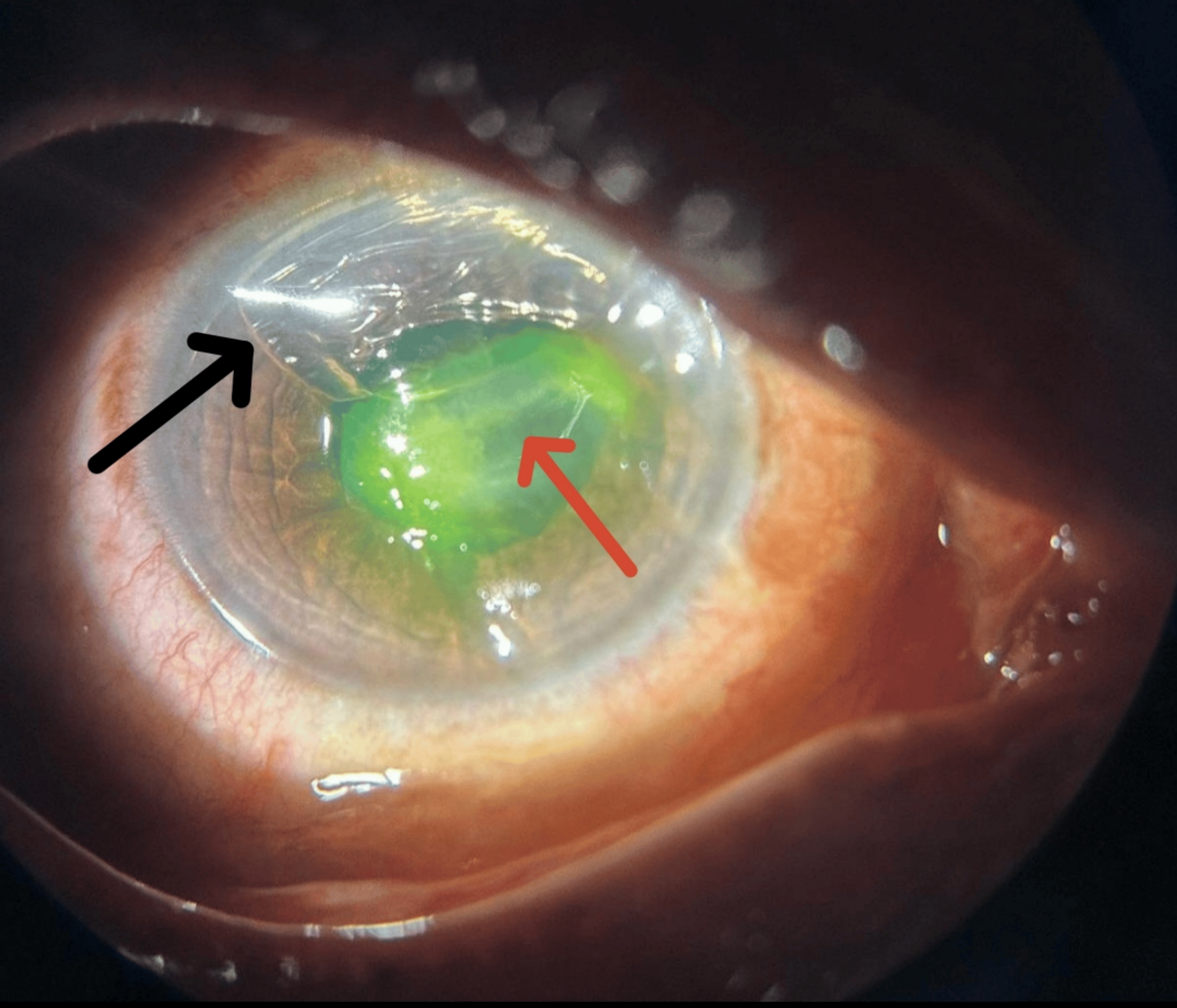
Slit-lamp photograph of the right eye showing the corneal perforation site (red arrow) and an intracameral air bubble (black arrow)

On examination, visual acuity in the right eye was perception of light with accurate projection of rays, while the left eye had a visual acuity of 6/9. Intraocular pressure (IOP) was not measured because globe perforation was suspected. Slit-lamp examination of the right eye revealed mild corneal edema with a central corneal defect. A distinct trapped air bubble was noted within the anterior chamber, with no anterior chamber reaction (Figure 1). The Seidel test was negative, and no iris prolapse, iris plugging, or obvious external wound gape was observed. The anterior chamber was relatively shallow. A traumatic cataract with disruption of the anterior lens capsule was present.

The presence of an intracameral air bubble in the setting of blunt trauma suggested an open-globe injury that may have spontaneously sealed prior to presentation. The corneal injury appeared consistent with a full-thickness perforation secondary to countercoup forces generated by blunt impact from the stone.

Posterior segment evaluation was limited because of hazy media. B-scan ultrasonography demonstrated an attached retina with vitreous echoes and no intraocular foreign body. Imaging of the orbit was not performed.

**Figure 2:**
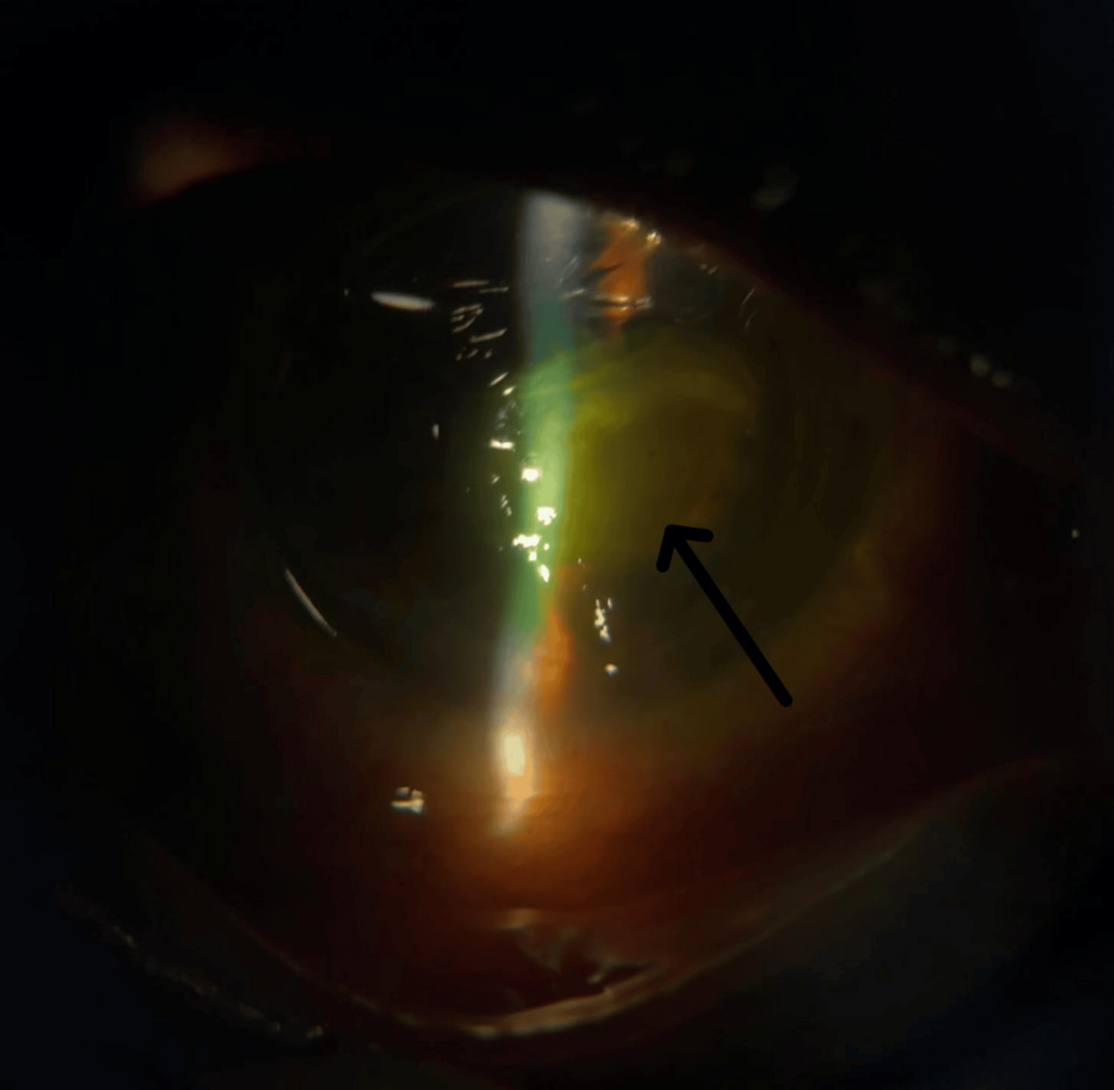
Slit-lamp optical section of the right eye showing a traumatic cataract with anterior lens subluxation

Slit-lamp optical sectioning further demonstrated a traumatic cataract with anterior lens subluxation (Figure 2). A diagnosis of self-sealed open-globe injury with corneal perforation and traumatic anterior lens subluxation with cataract was made. The patient was started on topical broad-spectrum antibiotics, cycloplegics, systemic antibiotics, and analgesics.

**Figure 3:**
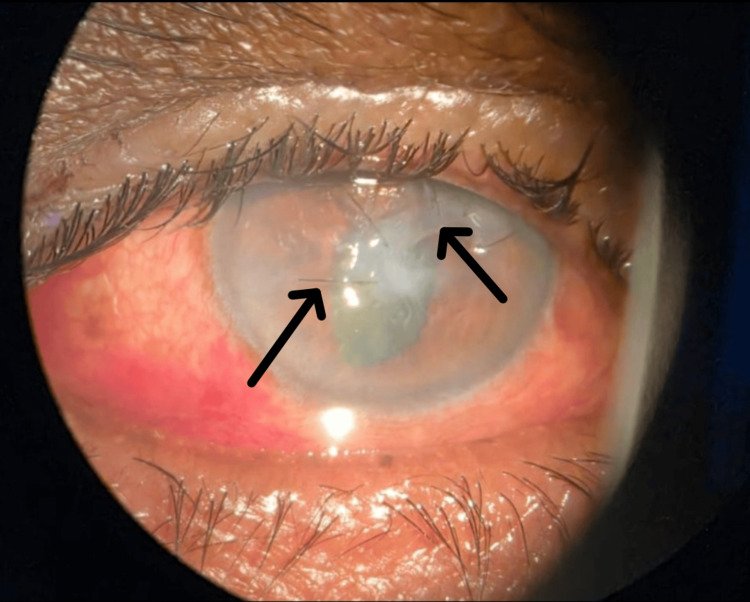
Follow-up anterior segment image of the right eye after corneal perforation repair

Considering the complexity of the anterior segment injury and the self-sealed globe perforation, a staged surgical approach was planned. Initial management involved meticulous repair of the central corneal tear, which measured approximately 8 mm and had a curved configuration, with suturing to restore globe integrity. Following the first intervention, restoration of globe integrity was achieved, with the resolution of corneal edema (Figure 3). One month after the primary surgery, lens extraction was performed for the traumatic cataract associated with a disrupted anterior lens capsule and prolapsed lens material. There was no vitreous in the anterior chamber. Secondary visual rehabilitation was subsequently achieved with scleral-fixated intraocular lens (SFIOL) implantation.

Postoperatively, the patient demonstrated gradual resolution of corneal edema and intraocular inflammation, with maintained anatomical integrity of the globe. At final follow-up, the patient achieved a best-corrected visual acuity of counting fingers at 3 meters, representing significant visual recovery considering the severity and deceptive nature of the initial presentation.

## Discussion

Ocular trauma accounts for about 3% of ophthalmic emergencies and is also an important cause of unilateral diminution of vision and permanent visual loss [3,4]. Blunt ocular trauma can occasionally result in hidden open-globe injuries that may initially masquerade as closed-globe trauma, particularly when the wound undergoes spontaneous sealing. Penetrating trauma is more common in young adults, whereas blunt trauma is more common in the elderly age group [5]. Corneal perforations following blunt trauma are uncommon because the cornea generally dissipates traumatic forces across the globe; however, severe focal impact may produce partial- or full-thickness corneal rupture through countercoup mechanisms [6]. In the present case, the absence of an obvious penetrating foreign body, lack of iris prolapse, negative Seidel test, and the presence of an intracameral air bubble suggested the diagnosis of an open-globe injury. The Seidel sign is not sensitive but is specific for globe rupture. A false-negative Seidel sign may be present on rare occasions [7].

A striking and diagnostically significant finding in this patient was the presence of a trapped intracameral air bubble. In the absence of prior surgical intervention or external air injection, an intracameral air bubble strongly suggests communication between the anterior chamber and the external environment at some point during the injury [8]. This feature raised suspicion of a self-sealed corneal perforation despite the absence of active aqueous leakage. The wound likely sealed spontaneously because of stromal edema and apposition of corneal tissue following trauma. Such subtle presentations can easily be overlooked, potentially delaying definitive management and increasing the risk of endophthalmitis or secondary inflammatory complications.

The traumatic cataract observed in this patient was associated with a disrupted anterior lens capsule and probable anterior subluxation of the crystalline lens. This was likely secondary to zonular weakness or disruption caused by transmission of blunt countercoup forces through the globe following a stone injury. Traumatic cataract is frequently associated with anterior or posterior lens subluxation or dislocation, capsular rupture, corneal injury, vitreous prolapse into the anterior chamber, and varying degrees of iris or vitreous loss [9]. Traumatic cataracts may develop immediately or progressively, depending on the severity of lenticular and zonular damage [10]. In our patient, the acute-onset cataract with anterior displacement of the lens suggested significant anterior segment trauma despite the absence of overt capsular rupture or prolapse of lens matter into the anterior chamber.

The mechanism of injury in this case appears distinct from conventional penetrating trauma. The corneal defect was presumed to result from countercoup forces generated by blunt impact from the stone, producing localized corneal rupture without an overt lacerating mechanism. Similar mechanisms have been described in severe blunt ocular trauma, where sudden globe compression causes rupture at structurally weaker points [11].

B-scan ultrasonography played an important role in excluding posterior segment involvement because media haze precluded adequate fundus visualization. Early surgical intervention remains essential in suspected occult open-globe injuries to restore globe integrity, reduce inflammation, and prevent complications such as infectious endophthalmitis, phacolytic glaucoma, synechiae formation, and retinal pathology, including vitreous hemorrhage, retinal tears, holes, dialysis, detachment, and choroidal rupture [12].

The standard protocol followed for the management of such injuries is primary globe repair, followed by secondary cataract removal and then intraocular lens implantation once ocular stability is achieved [13].

This case emphasizes the importance of meticulous slit-lamp examination in all cases of blunt ocular trauma presenting with corneal edema, a shallow anterior chamber, traumatic cataract, or unexplained intracameral air bubbles. Even in the absence of a positive Seidel test, clinicians should maintain a high index of suspicion for self-sealed open-globe injuries.

## Conclusions

This case highlights a deceptive presentation of blunt ocular trauma in which a presumed self-sealed corneal perforation presented with traumatic cataract and a trapped intracameral air bubble despite a negative Seidel’s test. The presence of an isolated air bubble within the anterior chamber served as a critical clue suggesting prior globe breach and spontaneous wound closure. Recognition of such subtle signs is essential to avoid delayed diagnosis of occult open-globe injuries. Careful slit-lamp evaluation, appropriate imaging, and timely surgical management are crucial in preserving globe integrity and optimizing visual outcomes following severe blunt ocular trauma.
